# Performance of a novel melting curve-based qPCR assay for malaria parasites in routine clinical practice in non-endemic setting

**DOI:** 10.1186/s12936-023-04617-z

**Published:** 2023-06-22

**Authors:** Kim J. M. van Bergen, Antoine R. Stuitje, Robert C. Akkers, Henricus J. Vermeer, Rob Castel, Theo G. Mank

**Affiliations:** 1 Result Laboratorium, Albert Schweitzer Hospital, Albert Schweitzerplaats 25, 3300 AK Dordrecht, The Netherlands; 2 MRC Holland, Willem Schoutenstraat 1, 1057 DL Amsterdam, The Netherlands; 3 Regional Laboratory for Medical Microbiology and Public Health, Boerhaavelaan 26, 2035 RC Haarlem, The Netherlands

**Keywords:** Malaria, *Plasmodium*, qPCR, Melting curve analysis, Therapy-monitoring, Quantification parasitaemia

## Abstract

**Background:**

High-quality malaria diagnosis is essential for effective treatment and clinical disease management. Microscopy and rapid diagnostic tests are the conventional methods performed as first-line malaria diagnostics in non-endemic countries. However, these methods lack the characteristic to detect very low parasitaemia, and accurate identification of the *Plasmodium* species can be difficult. This study evaluated the performance of the MC004 melting curve-based qPCR for the diagnosis of malaria in routine clinical practice in non-endemic setting.

**Methods and results:**

Whole blood samples were collected from 304 patients with clinical suspicion of malaria and analysed by both the MC004 assay and conventional diagnostics. Two discrepancies were found between the MC004 assay and microscopy. Repeated microscopic analysis confirmed the qPCR results. Comparison of the parasitaemia of nineteen *Plasmodium falciparum* samples determined by both microscopy and qPCR showed the potential of the MC004 assay to estimate the parasite load of *P. falciparum*. Eight *Plasmodium* infected patients were followed after anti-malarial treatment by the MC004 assay and microscopy. The MC004 assay still detected *Plasmodium* DNA although no parasites were seen with microscopy in post-treatment samples. The rapid decline in *Plasmodium* DNA showed the potential for therapy-monitoring.

**Conclusion:**

Implementation of the MC004 assay in non-endemic clinical setting improved the diagnosis of malaria. The MC004 assay demonstrated superior *Plasmodium* species identification, the ability to indicate the *Plasmodium* parasite load, and can potentially detect submicroscopic *Plasmodium* infections.

**Supplementary Information:**

The online version contains supplementary material available at 10.1186/s12936-023-04617-z.

## Background

Malaria is a life-threatening infectious disease caused by *Plasmodium* parasites transmitted by infected *Anopheles* mosquitoes in (sub)tropical areas. The World Health Organization (WHO) considers malaria a leading public health concern due to its potential lethal complications. In 2021, the WHO estimated 247 million malaria cases, leading to 619,000 malaria deaths worldwide [1]. Although progress has been made in global malaria control, malaria remains a threat, not only to the population in endemic areas, but also to travellers and migrants [2]. Among the seven species that infect humans, *Plasmodium falciparum* is responsible for the majority of malaria deaths worldwide [1, 3].

Early and accurate diagnosis of malaria is essential for both effective clinical management and malaria control. High-quality malaria diagnosis is important, as misdiagnosis can result in significant morbidity and mortality [4, 5]. In non-endemic countries, a combination of microscopic examination (thick and thin blood smears) and a rapid diagnostic test (RDT) is commonly used as first-line malaria diagnosis.

Microscopic examination of Giemsa-stained thick and thin blood smears is the most widely used method, and remains the gold standard for the diagnosis of malaria. Microscopic examination provides inexpensive and rapid detection and identification of the *Plasmodium* species, stage levels, and allows determination of the parasite density (parasitaemia). The major limitations of microscopy are the need for experienced microscopists, and decreased accuracy when the parasite density is low [6, 7]. Misdiagnosis has been reported frequently, as distinction between *Plasmodium* species can be difficult or even impossible and the limit of detection (LoD) of the Giemsa-stained thick blood smear (5 to 50 parasites/μL of blood) is not always sufficient to detect low-density infections [6, 8, 9].

RDTs are immunochromatographic tests that are inexpensive, easy to use and results can be obtained within 5 to 15 min. The most widely used RDTs are based on the detection of histidine-rich protein II (HRP-II) of *P. falciparum* and lactate dehydrogenase or aldolase common to all *Plasmodium* species. RDTs have reasonable sensitivity to detect *P. falciparum*, but lack sensitivity for the detection of other *Plasmodium* species [10, 11]. The limit of detection of RDTs is in the range of 200 to 2000 parasites per μL of blood [12]. Another limitation of RDTs is the occurrence of false-negative results due to deletions of the HRP-II gene in some *P. falciparum* strains [13].

To overcome the limitations of microscopy and RDTs, molecular assays based on the detection of *Plasmodium* DNA have been proposed as a confirmatory method. Several nucleic acid amplification tests (NAATs) have been developed for the detection of *Plasmodium* DNA. These include conventional polymerase chain reaction (PCR), qPCR and loop-mediated isothermal amplification tests (LAMP), which mostly detect genus- or species-specific DNA-sequences of the *Plasmodium* parasite [14, 15]. NAATs allow superior species identification and are at least tenfold more sensitive than microscopy [16–21]. The LoD of NAATs is between 0.002 to 6 parasites per μL of blood, depending on the type of NAAT and the *Plasmodium* species [18–21]. In addition, NAATs that allow quantification of the parasitaemia to make clinical management decisions are described in literature [22, 23].

However, a well-known issue with NAATs is the possible occurrence of cross-reactivity between different *Plasmodium* species. Especially the nucleotide sequences of *Plasmodium vivax*, *Plasmodium knowlesi* and *Plasmodium cynomolgi* share high similarity, which may cause misidentification and mistreatment [24, 25]. Furthermore, NAATs have been reported to detect *Plasmodium* DNA up to several weeks after effective anti-malarial treatment, potentially leading to over-diagnosis of recrudescence [26]. The MC004 melting curve-based qPCR assay was recently developed to detect, quantify and discriminate between *P. falciparum*, *P. vivax*, *Plasmodium malariae*, *Plasmodium ovale wallikeri*, *Plasmodium ovale curtisi*, *P. knowlesi* (including differentiation of three strains) and *P. cynomolgi* (including differentiation of three strains) [27].

Furthermore, a prospective cross-sectional study was performed in malaria endemic setting (central Ethiopia) to further assess the performance of MC004 for the detection and identification of *Plasmodium* parasites compared to standard microscopy [28]. The present study was designed to evaluate the performance of the MC004 assay in routine clinical practice in a non-endemic setting. Accuracy of the MC004 assay for malaria diagnosis in blood samples from patients with clinical suspicion of malaria was determined by comparison to the standard method of microscopy and RDT. In addition, follow-up of malaria treated patients was assessed.

## Methods

### Study population

The performance of the MC004 assay (melting curve-based qPCR) in routine clinical practice was determined by analysing clinical samples received from different laboratories (Result Laboratory—Dordrecht (Albert Schweitzer hospital), the Netherlands, Breda (Amphia hospital), the Netherlands, Regional Laboratory for Medical Microbiology & Public Health—Haarlem, the Netherlands, Noordwest Ziekenhuisgroep—Alkmaar, the Netherlands). In total, 318 ethylenediaminetetraacetic acid (EDTA) whole blood samples obtained from 304 patients with clinical suspicion of malaria were included in the study. All patients had travelled to malaria-endemic areas and presented with signs and symptoms of malaria. A series of post-malaria treatment samples (one to four samples per patient) from five *P. falciparum* infected patients; one *P. vivax,* one *P. malariae* and one *P. ovale* were available and also included. These follow-up samples were used to examine the correlation between the RFU (Relative Fluorescence Units) of the melting curves determined by the MC004 assay and parasite density (parasitaemia) determined by microscopy. The follow-up samples were not collected systematically, but determined based on the clinician’s request for laboratory testing.

All blood samples were taken as part of routine blood sampling and examined by microscopy (Giemsa-stained thick and thin blood smear) and a rapid diagnostic test (RDT). The MC004 assay was performed blindly (retrospectively) after the initial diagnosis by microscopy. For the follow-up patients, only the first sample had been examined by all available diagnostic tests; RDT, microscopy and the MC004 assay. The results of the MC004 assay were compared with the results by microscopy.

### Rapid diagnostic testing

The Palutop + 4 Optima (Biosynex, Strasbourg, France) immunochromatographic test (ICT) was used for rapid diagnostic testing of the clinical samples from Result Laboratory according to the manufacturer’s instructions. The test can detect and differentiate between *P. falciparum* by targeting Histidine-Rich Protein II (HRP-II), *P. vivax* by *P. vivax*-specific parasite lactate dehydrogenase (Pv-pLDH) and pan-pLDH for *Plasmodium* species [6]. The BinaxNOW malaria RDT (Binax Inc, Inverness Medical, ME, USA) was used by the Regional Laboratory for Medical Microbiology & Public Health, Noordwest Ziekenhuisgroep and Amphia hospital for the detection of the HRP-II antigen specific to *P. falciparum* and pan-aldolase for *Plasmodium* species, following manufacturer’s instructions [6].

### Microscopy

The thick and thin blood smears were stained with 3% Giemsa in phosphate-buffer (pH 7.2), followed by microscopic examination (1000 × magnification) by two experienced technicians, according to WHO recommendations [29]. A blood smear was defined as “No malaria parasites seen” if a minimum of 100 fields were examined. If parasites were detected, the thin blood smears were used to identify the *Plasmodium* species and in the case of *P. falciparum* or *P. knowlesi* also the parasitaemia was calculated, following the Dutch standard guidelines for thin smear microscopy [30, 31].

### DNA extraction

DNA was extracted from 200 µL of (EDTA) human whole blood using the QIAamp DNA Blood Mini QIAcube Kit (Qiagen, Hilden, Germany) on a QIAcube instrument (Qiagen, Hilden, Germany), following the manufacturer’s specifications. DNA was eluted with 100 µL elution buffer and the processed specimen samples were stored at − 30 °C.

### MC004 melting curve-based qPCR

The previously described MC004 assay (MRC-Holland, Amsterdam, the Netherlands) is a single tube multiplex qPCR for the detection and identification of *Plasmodium* species that cause malaria in humans [27]. The assay targets the mitochondrial DNA of *Plasmodium* species and discriminates between 11 *Plasmodium* species/strains (*P. falciparum*, *P. vivax*, *P. malariae*, *P. ovale wallikeri*, *P. ovale curtisi*, *P. knowlesi* LT48, *P. knowlesi* ATCC 30153, *P. knowlesi* ATCC 30158 *P. cynomolgi* ATCC 30149, *P. cynomolgi* KJ569866.1 and *P. cynomolgi* KJ569868.1). The MC004 assay involves two main steps. (1) asymmetric target amplification by two different primer sets, primer pair 1 designed to amplify all 11 *Plasmodium* species/strains and primer pair 2 designed to amplify only *P. vivax*, *P. knowlesi*, and *P. cynomolgi*. (2) detection and differentiation of the amplicons using probe-based melting curve analysis. The three different molecular beacon probes were labelled with either Texas Red, Cy5, or Cy5.5. The PCR reaction was performed in a final volume of 25 µL, including 23 µL MC004 Mastermix (MRC-Holland, Amsterdam, the Netherlands) and 2 µL of extracted DNA. Amplification was performed using the CFX96 Touch Real-time PCR Detection System (Bio-Rad, Hercules, CA, USA) and the following settings: 95 °C for 3 min, 50 cycles of 95 °C for 15 s, 60 °C for 30 s and 68 °C for 40 s. Followed by a melting curve step: gradual temperature increase from 25 to 69.4 °C (0.4 °C per 5 s).

Positive and negative controls were used to monitor run validity. Each run included two negative controls: elution buffer added as template and a fresh uninfected human whole blood specimen processed as a separate sample. This control should produce an amplification curve, if no amplification curve was observed, this may be a sign of inhibition and makes the result invalid. To guarantee the analytical sensitivity of the assay, every run also included a positive control for limit of detection (1st WHO International Standard for *P. falciparum* DNA, diluted in EDTA whole blood to a concentration of 1 × 10^–3^ IU/mL) [32]. Positive controls for each *Plasmodium* species come with the MC004 assay as provided by MRC Holland.

### Quantification of the parasitaemia by the MC004 assay

Nineteen positive *P. falciparum* samples were used to examine the association between the Cq-value determined with the MC004 assay and the parasitaemia determined by thin blood smear microscopy. The parasitaemia based on Cq-value was calculated using the previously described calibration curve of the MC004 assay [27]. For the MC004 assay, a Cq-value > 21.7 was reported as a parasitaemia of < 0.1%.

### Statistical analysis

Linear regression analysis and calculation of R-squared were performed using Microsoft Excel for Mac 2016. Confidence intervals and P-values were calculated using GraphPad Prism 9 for macOS (version 9.5.1), modules ‘Simple linear regression’ and ‘Correlation’.

## Results

### Clinical performance of the MC004 assay compared to microscopy and RDT

A total of 304 patients were included in the study for the comparison between microscopy, RDT and the MC004 assay. As shown in Table 1, the MC004 assay tested positive in 34 cases, including 27 *P. falciparum*, three *P. vivax*, two *P. malariae*, one *P. ovale wallikeri* and one *P. ovale curtisi*. No *P. knowlesi*, *P. cynomolgi* or mixed infections were detected in the examined patients. The remaining 270 patients tested negative for the presence of *Plasmodium* DNA. Twenty-seven (100%) of 27 *P. falciparum* PCR positive samples were identified by microscopy, with parasitaemias ranging from < 0.1% to 35.8%. *P. vivax* was identified in 2 of 3 cases (67%), *P. malariae* in 2 of 2 cases (100%) and *P. ovale* in 2 of 2 cases (100%) of the PCR positive samples (see Table 1). However, one PCR positive *P. falciparum* sample was identified as mixed infection of *P. falciparum* and *P. vivax* by microscopy. RDT tested positive in all 27 PCR positive *P. falciparum* samples, but missed all PCR-positive *P. vivax*, *P. malariae* and *P. ovale* cases.

**Table 1:** Agreement between microscopy, RDT and the MC004 assay for patients with suspected malaria

Microscopy		Melting curve-based qPCR (MC004)					RDT	RDT
	*Pf*	*Pv*	*Pm*	*Pow*	*Poc*	Negative	( +)	(−)
*Pf*	26	0	0	0	0	0	26	0
*Pv*	0	2	0	0	0	0	0	3
*Pm*	0	0	2	0	0	0	0	2
*Po*	0	0	0	1	1	0	0	2
Mixed	1*	0	0	0	0	0	1**	0
No malaria parasites seen	0	**1**	0	0	0	270	0	270
Total	27	3	2	1	1	270	27	277

Note the two discrepancies in bold

*Pf/Pv/Pm/Pow/Poc/Po* = *P. falciparum*, *P. vivax*, *P. malariae*, *P. ovale wallikeri*, *P. ovale curtisi*, *P. ovale*

*Misidentified mixed infection of *P. falciparum* + *P. vivax*

**Presence *P. falciparum*, *P. vivax* and PAN band

Analysis of the data showed two discrepancies. In one *P. vivax* PCR positive sample, *P. vivax* parasites were not seen with microscopy and the sample was also tested negative with RDT (Palutop + 4 Optima). However, after blind re-examination of the blood smear *P. vivax* trophozoites were seen. Furthermore, a mixed infection of *P. falciparum* and *P. vivax* was identified by microscopy, whereas the MC004 assay only detected the presence of *P. falciparum* DNA. The RDT showed the presence of the HRP-II, Pv-pLDH and pan-pLDH band (Palutop + 4 Optima). Blind re-examination of the blood smear identified the presence of only *P. falciparum* trophozoites (parasitaemia 1.3%). Therefore, both sensitivity and specificity were 100% for the MC004 assay compared to microscopy.

### Follow-up during anti-malarial treatment with the MC004 assay

**Fig. 1:**
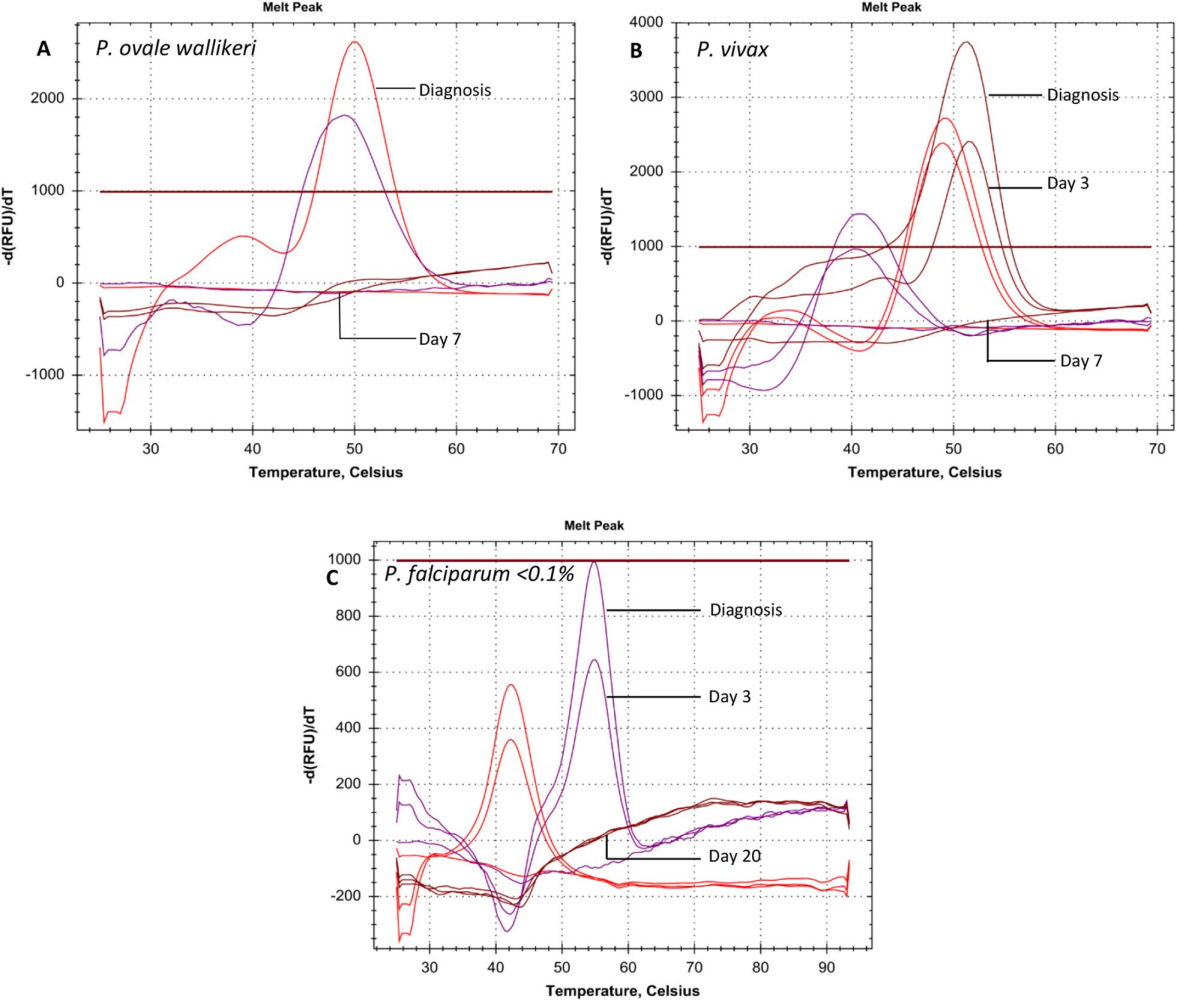
Melting curves of one *P. ovale wallikeri* (**A**), one *P. vivax* (**B**) and one *P. falciparum* (parasitaemia < 0.1%) (**C**) patient followed after starting anti-malarial treatment. The specific melting curve pattern was used to identify the *Plasmodium* species. The x-axis shows the temperature (°C). The y-axis shows the negative derivative of fluorescence (RFU) with respect to temperature (T). The *Plasmodium* species is indicated in the left corner of each figure. The amount of days after the start of anti-malarial treatment is indicated at the right of the melting curves. Red curves correspond to the Texas Red labelled probe, purple curves to the Cy5 labelled probe, and brown curves to the Cy5.5 labelled probe. The MC004 assay was reported as negative if no melting curves were present. For the sake of clarity, not all follow-up samples are included in the figures

Five *P. falciparum* infected patients (parasitaemia ranging from < 0.1 to 3.1%), one *P. vivax*, one *P. malariae* and one *P. ovale wallikeri* infected patient were followed after starting anti-malarial treatment. The follow-up samples (one to four samples per patient) were examined by microscopy and the MC004 assay. In one *P. falciparum* (parasitaemia < 0.1%), one *P. vivax* and one *P. ovale wallikeri* infected patient, the MC004 assay became negative during follow-up (no melting curves detected). In all three cases, no malaria parasites were seen with microscopy although malaria DNA was still detected by the MC004 assay, see Table 2. Melting curves were absent 20 days after the start of anti-malarial treatment for *P. falciparum* and after 7 days of treatment for *P. vivax* and *P. ovale wallikeri,* shown in Fig. 1A–C.

**Table 2:** Microscopy versus the MC004 assay in follow-up patients after start anti-malarial treatment

*Plasmodium*-species		Days after start anti-malarial treatment						
		Day 1	Day 2	Day 3	Day 7	Day 8	Day 12	Day 20
*P. falciparum* 3.1%	Microscopy					−		
	MC004 assay					+		
*P. falciparum* 0.2%	Microscopy						−	
	MC004 assay						+	
*P. falciparum* 0.1%	Microscopy			−				
	MC004 assay			+				
*P. falciparum* < 0.1%	Microscopy	−		−				−
	MC004 assay	+		+				−
*P. vivax*	Microscopy	+	−	−	−			
	MC004 assay	+	+	+	−			
*P. ovale*	Microscopy				−			
	MC004 assay				−			
*P. malariae*	Microscopy			−				
	MC004 assay			+				

+   Positive, − Negative

The follow-up of the other five patients (one *P. malariae* and four *P. falciparum*) stopped when no parasites were seen anymore with microscopy (Table 2). The MC004 assay did not become negative, but a decrease in RFU of the melting curves was observed in every patient, see Additional file 1: Fig. S1. Decreased melting curves were observed 12 days after the start of anti-malarial treatment for *P. falciparum* (parasitaemia 0.2%)*,* 8 days for *P. falciparum* (parasitaemia 3.1%) and 3 days for *P. malariae*, *P. falciparum* (parasitaemia 0.1%) and *P. falciparum* (parasitaemia 0.1%).

### Comparison of calculated parasitaemia by the MC004 assay and microscopy

**Fig. 2:**
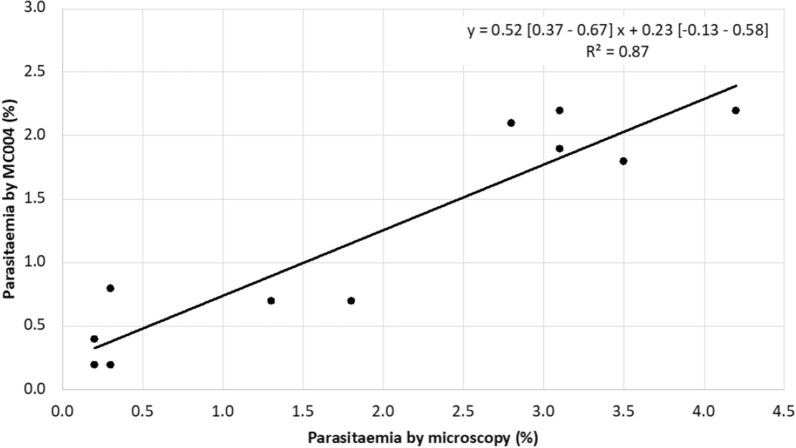
Statistical analysis of parasitaemia determined by microscopy and the MC004 assay. The solid black line represents the fitted linear regression line of which the equation is shown in the upper-right corner of the graph. The 95% confidence intervals of the best-fit values of the slope and Y-intercept are shown in brackets. The slope is significantly non-zero (P-value < 0.0001). R-squared (0.87; P-value < 0.0001) is also shown in the upper right corner. Values preceded by a 'less than' symbol ( <) were excluded

The parasitaemia of nineteen *P. falciparum* samples was determined by microscopy and calculated using the calibration curve of the MC004 assay. The results of the MC004 assay were compared with the results by microscopy, shown in Table 3. The parasitaemia levels determined by the MC004 assay are highlighted in red if the value was outside the range of the 95% confidence interval of the parasitaemia levels determined by microscopy [16]. The range of absolute differences was − 2.0% (at parasitaemia level of 4.2%) to 0.5% (at parasitaemia level of 0.3%). In none of the nineteen samples schizonts or gametocytes were seen by microscopy. Figure 2 shows the graphical comparison between parasitaemia estimated by microscopy and the MC004 assay. A linear regression line was fitted (parasitaemia by MC004 equals 0.52 times the parasitaemia by microscopy plus 0.23) and R-squared was calculated (0.87).

**Table 3:** Comparison of microscopy and MC004 assay for the quantification of parasitaemia

Parasitaemia (%) [95% confidence interval] microscopy	Parasitaemia (%) MC004 assay	Cq-value	Difference (%) (P_MC004_-P_Mic_)
4.2 [3.0–5.4]	**2.2**	16.56	− 2.0
3.5 [2.4–4.6]	**1.8**	16.90	− 1.7
3.1 [2.0–4.2]	2.2	16.55	− 0.9
3.1 [2.0–4.2]	**1.9**	16.76	− 1.2
2.8 [1.8–3.8]	2.1	16.61	− 0.7
1.8 [1.0–2.6]	**0.7**	18.36	− 1.1
1.3 [0.6–2.0]	0.7	18.30	− 0.6
0.4 [0.0–0.8]	< 0.1	22.13	Not applicable
0.3 [0.0–0.6]	**0.8**	18.17	0.5
0.3 [0.0–0.6]	0.2	20.42	− 0.1
0.2 [0.0–0.5]	0.4	19.38	0.2
0.2 [0.0–0.5]	0.2	20.42	0
0.1 [0.0–0.3]	< 0.1	22.13	Not applicable
< 0.1 [Not applicable]	0.1	21.25	Not applicable
< 0.1 [Not applicable]	0.1	21.40	Not applicable
< 0.1 [Not applicable]	< 0.1	21.91	Not applicable
< 0.1 [Not applicable]	< 0.1	23.35	Not applicable
< 0.1 [Not applicable]	< 0.1	24.34	Not applicable
< 0.1 [Not applicable]	< 0.1	29.04	Not applicable

MC004 parasitaemia levels outside the 95% confidence interval (determined by microscopy) are in bold [16]

P = Parasitaemia

## Discussion

### Clinical performance of the MC004 assay compared to microscopy and RDT

In this study, the clinical performance of the novel MC004 melting curve-based qPCR assay compared to microscopy and RDT was evaluated for the detection and identification of *Plasmodium* parasites. The MC004 assay detects *Plasmodium* mitochondrial DNA from *Plasmodium* parasites that cause malaria in humans, and identifies the *Plasmodium* species using melting curve analysis based on three different molecular beacon probes. The technical validation of the MC004 assay using reference samples and synthetic controls was previously reported [27].

In routine non-endemic setting, the MC004 assay showed 100% sensitivity and specificity for the diagnosis of malaria in humans. These results align with the findings reported by Beyene et al. [28], which demonstrated a 100% sensitivity of the MC004 assay compared to microscopy. The specificity was slightly lower at 96.7% due to the misidentification of mixed infections as single infections by the MC004 assay.

Here, two discrepant results were observed between the MC004 assay and microscopy. One PCR positive *P. vivax* was missed by microscopy and one PCR positive *P. falciparum* was misidentified as a mixed infection of *P. falciparum* and *P. vivax* (Table 1). Both discrepant results could be related to the result of the RDT, since technicians performing the microscopic examination were biased by the RDT results.

The most likely explanation for the missed *P. vivax* is inadequate microscopic examination, as result of a negative RDT result, although it is known that RDTs can give false-negative results in case of non-*falciparum* infections. Since no parasites were reported during the first examination in combination with a negative RDT result, the possibility of malaria was deemed to be highly unlikely by the second microscopist, who consequently also failed to identify *P. vivax* parasites. The initial misdiagnosis delayed the treatment of this patient by nine months, highlighting the important diagnostic value of qPCR as additional test in the diagnosis of malaria. Misdiagnosis in the case of non-*falciparum* malaria is not uncommon in non-endemic setting and raised as a concern in previously reported literature [33]. RDTs mainly focus on the diagnosis of *P. falciparum* and maintaining expertise in the microscopic diagnosis is difficult, as each single laboratory in non-endemic setting only encounters a small number of malaria cases per year.

In the case of the misidentified mixed infection by microscopy, the RDT showed the presence of the *P. falciparum* (HRP-II), *P. vivax* (Pv-pLDH), and pan-pLDH band (Palutop + 4 Optima). Although cross-reaction of *P. falciparum* with the *P. vivax* Pv-pLDH antigen is a known issue for RDTs, misunderstanding of the RDT bands could have influenced the microscopic examination [34, 35]. Blind re-examination of the blood smears showed that the result of the MC004 assay regarding this discrepancy was also correct; only *P. falciparum* parasites were seen.

No *P. knowlesi*, *P. cynomolgi* or mixed infections were detected by the MC004 assay during this study, indicating that these infections are rare in non-endemic setting. The same observation was reported by Gier et al. for the period of 2008–2015 in the Netherlands and Calderaro et al. for the period of 2013–2017 in Italy [36, 37].

Regarding the performance of the RDT, the results highlight that the use of RDTs is useful especially for the diagnosis of *P. falciparum*, although, in order to ensure correct interpretation, their limitations should be taken into account. Besides the already mentioned limitation, one should be well aware of the fact that *P. falciparum* variants are circulating that lack HRP-II. Correct identification of these *P. falciparum* variants by HRP-II-based assays may be compromised [38].

### Follow-up during anti-malarial treatment with the MC004 assay

In the present study, several follow-up samples were obtained from eight different patients during anti-malarial treatment. The MC004 assay could still detect *Plasmodium* DNA although no parasites were seen anymore with microscopy. The MC004 assay became negative within 7 days after starting anti-malarial treatment for one *P. vivax* and one *P. ovale* infected patient, and between day 4 and 20 for a *P. falciparum* infected patient (Table 2, Fig. 1A–C). The five remaining patients showed decreased melting curves after starting anti-malarial treatment (Additional file 1: Fig. S1), also indicating the process of parasite clearance.

A higher parasite clearance rate was expected for the patients with a low parasitaemia (≤ 0.2%) compared to the patient with a high parasitaemia (3.1%), since less *Plasmodium* DNA would be present. However, the difference in parasite clearance rate could not be correlated with the initial parasite density or with the presence of gametocytes pre-treatment (determined by microscopy). A *P. falciparum* infected patient with a parasitaemia of 3.1% showed melting curves around 1000 RFU at day 8 after starting anti-malarial treatment, whereas the same RFU was observed at day 12 for a *P. falciparum* infected patient with a parasitaemia of only 0.2%. Other factors that could have influenced the parasite clearance rate are the drug concentration, drug resistance and host malaria-specific immunity [39, 40]. According to the Dutch guideline for malaria diagnostics, PCR is not suitable for determining the parasitaemia in follow-up patients, since PCR cannot distinguish between DNA originating from asexual and sexual lifecycle stages, and DNA originating from viable and non-viable parasites [41]. Nevertheless, a decrease in RFU of the melting curves for each patient receiving anti-malarial treatment was shown.

One additional benefit of the MC004 assay is its high negative predictive value. According to the UK guideline, the microscopic examination should be repeated three times (every 12–24 h) in order to rule out the diagnosis of malaria [42]. Since over 80% of all malaria tests performed for patients suspected of imported malaria are negative, the MC004 assay can greatly reduce the need for repeated microscopic examination [43]. In addition, the Dutch guideline reported that a negative PCR 28 days after anti-malarial-treatment can rule out recrudescence or relapse in follow-up patients [41].

### Quantification of the parasitaemia by the MC004 assay compared to microscopy

Nineteen *P. falciparum* samples were used to evaluate the calculated parasitaemia by the MC004 assay with the parasitaemia determined with microscopy. In fourteen of the nineteen samples the parasitaemia levels determined by the MC004 assay were inside the 95% confidence intervals of the parasitaemia levels determined by microscopy. In five samples, including the two highest parasitaemia levels of 4.2% and 3.5%, the parasitaemia levels determined by the MC004 assay fell outside the confidence interval (Table 3) [16]. Thus, the MC004 assay underestimated a parasitaemia of over 4%, which is a threshold to indicate severe malaria [49]. Figure 2 shows that the parasitaemia estimated by the MC004 assay and by microscopy were strongly correlated (R-squared of 0.87), and the tendency of the MC004 assay to underestimate the parasitaemia compared to microscopy.

In microscopy only the trophozoite stages are counted and an erythrocyte infected with multiple trophozoites is counted as one infected erythrocyte. The parasitaemia calculated by the MC004 assay was mostly lower than the parasitaemia determined with microscopy. Since the MC004 assay cannot distinguish between DNA originating from the different lifecycle stages, overestimation of parasitaemia by the MC004 assay might have been expected rather than underestimation. However, in none of the samples schizonts or gametocytes were seen by microscopy. Therefore, the effect of the presence of schizonts and gametocytes remains unknown. Furthermore, the limited number of samples hinders certainty regarding whether the observed differences in parasitemia represent systematic bias or variation. Large variation in parasitaemia determined by microscopy is a well-known phenomenon [44]. Microscopy is the gold standard for determining the parasitaemia, however, the accuracy of microscopy is influenced by the technician’s expertise, choice of film type (thick or thin smear), the amount of examined fields and red blood cell count [45, 46]. On the other hand, small differences in Cq-value can result in large differences in the calculated parasitaemia by the MC004 assay, especially in the case of high parasitaemia (low Cq-value).

This study showed that the MC004 assay has potential for indication of parasitaemia in uncomplicated *P. falciparum* malaria (parasitaemia < 4%). Further research would be needed to evaluate quantification of parasitaemia in severe or complicated malaria (> 4%).

### Limitations of the study

The present study had limitations. During the study period, no mixed infection were observed, as mixed infections are rare in non-endemic setting [36, 37]. Therefore, the ability of the MC004 assay for the detection of mixed infections in clinical samples could not be evaluated. Another limitation concerns the lack of systematically collected follow-up samples for each patient and the small sample size of eight follow-up patients. Furthermore, quantification of samples with high parasitaemias (> 4%) by the MC004 assay could not be evaluated thoroughly, since only a single sample with a parasitaemia > 4% was present during this study. In addition, the diagnostic performance of the MC004 assay should be compared to that of alternative molecular diagnostic assays, in particular LAMP assays, which are becoming increasingly used in developed countries [47, 48].

## Conclusion

The MC004 melting curve-based qPCR assay showed 100% sensitivity and specificity for the diagnosis of malaria within routine non-endemic setting. Patient health care and clinical disease management were improved with the implementation of the MC004 assay, especially in the case of non-*falciparum* malaria. The MC004 assay demonstrated more accurate *Plasmodium* species identification, the ability to indicate the parasite load of *P. falciparum*, and can potentially detect submicroscopic *Plasmodium* infections. In addition, this study indicated the potential of the MC004 assay for therapy-monitoring reflected by the RFU of melting curves.

## Supplementary Information

**Additional file 1: Fig S1.** Melting curves of one *P. malariae* patient and four *P. falciparum* (parasitaemia ranging from 0.1-3.1%) patients that were followed after starting anti-malarial treatment. The specific melting curve pattern was used to identify the *Plasmodium* species. The x-axis shows the temperature (°C). The y-axis shows the negative derivative of fluorescence (RFU) with respect to temperature (T). The *Plasmodium* species is indicated in the left corner of each figure. The amount of days after the start of anti-malarial treatment is indicated at the right of the melting curves. Red curves correspond to the Texas Red labelled probe, purple curves to the Cy5 labelled probe, and brown curves to the Cy5.5 labelled probe. For the sake of clarity, not all follow-up samples are included in the figures.

## Data Availability

All datasets are presented in the manuscripts. Raw data of the qPCR-runs and patient materials are not publicly available due to patient privacy concerns, but on reasonable request to the corresponding author every effort will be made to answer the questions raised.

## References

[1] WHO. World malaria report 2022. Geneva, World Health Organization, 2022. https://apps.who.int/iris/handle/10665/365169. Accessed 9 Aug 2022.

[2] Garcia-Ruiz de Morales A, Morcate C, Isaba-Ares E, Perez-Tanoira R, Perez-Molina JA (2021). High prevalence of malaria in a non-endemic setting among febrile episodes in travellers and migrants coming from endemic areas: a retrospective analysis of a 2013–2018 cohort. Malar J.

[3] Sato S (2021). *Plasmodium* - a brief introduction to the parasites causing human malaria and their basic biology. J Physiol Anthropol.

[4] Berzosa P, de Lucio A, Romay-Barja M, Herrador Z, González V, García L (2018). Comparison of three diagnostic methods (microscopy, RDT, and PCR) for the detection of malaria parasites in representative samples from Equatorial Guinea. Malar J.

[5] Oyegoke OO, Maharaj L, Akoniyon OP, Kwoji I, Roux AT, Adewumi TS (2022). Malaria diagnostic methods with the elimination goal in view. Parasitol Res.

[6] Chiodini PL (2014). Malaria diagnostics: now and the future. Parasitology.

[7] Wongsrichanalai C, Barcus MJ, Muth S, Sutamihardja A, Wernsdorfer WH (2007). A review of malaria diagnostic tools: microscopy and rapid diagnostic test (RDT). Am J Trop Med Hyg.

[8] Coleman RE, Maneechai N, Rachaphaew N, Kumpitak C, Miller RS, Soyseng V (2002). Comparison of field and expert laboratory microscopy for active surveillance for asymptomatic *Plasmodium falciparum* and *Plasmodium vivax* in western Thailand. Am J Trop Med Hyg.

[9] McKenzie FE, Sirichaisinthop J, Miller RS, Gasser RA, Wongsrichanalai C (2003). Dependence of malaria detection and species diagnosis by microscopy on parasite density. Am J Trop Med Hyg.

[10] Krishna S, Bhandari S, Bharti PK, Basak S, Singh N (2017). A rare case of quadruple malaria infection from the highly malaria-endemic area of Bastar, Chhattisgarh, India. PLoS Negl Trop Dis.

[11] van Dijk DP, Gillet P, Vlieghe E, Cnops L, van Esbroeck M, Jacobs J (2009). Evaluation of the Palutop+4 malaria rapid diagnostic test in a non-endemic setting. Malar J.

[12] WHO. Results of WHO product testing of malaria RDTs: round 8 (2016–2018). Geneva, World Health Organization, 2018. https://www.who.int/publications/i/item/9789241504720. Accessed 11 Aug 2022.

[13] Parr JB, Verity R, Doctor SM, Janko M, Carey-Ewend K, Turman BJ (2017). Pfhrp2-deleted *Plasmodium falciparum* parasites in the Democratic Republic of the Congo: a national cross-sectional survey. J Infect Dis.

[14] Charpentier E, Benichou E, Pagès A, Chauvin P, Fillaux J, Valentin A (2020). Performance evaluation of different strategies based on microscopy techniques, rapid diagnostic test and molecular loop-mediated isothermal amplification assay for the diagnosis of imported malaria. Clin Microbiol Infect.

[15] Nijhuis RHT, van Lieshout L, Verweij JJ, Claas ECJ, Wessels E (2018). Multiplex real-time PCR for diagnosing malaria in a non-endemic setting: a prospective comparison to conventional methods. Eur J Clin Microbiol Infect Dis.

[16] Bailey JW, Williams J, Bain BJ, Parker-Williams J, Chiodini PL (2013). Guideline: the laboratory diagnosis of malaria. General Haematology Task Force of the British Committee for Standards in Haematology. Br J Haematol..

[17] Perandin F, Manca N, Calderaro A, Piccolo G, Galati L, Ricci L (2004). Development of a real-time PCR assay for detection of *Plasmodium falciparum*, *Plasmodium vivax,* and *Plasmodium ovale* for routine clinical diagnosis. J Clin Microbiol.

[18] Farrugia C, Cabaret O, Botterel F, Bories C, Foulet F, Costa JM (2011). Cytochrome b gene quantitative PCR for diagnosing *Plasmodium falciparum* infection in travelers. J Clin Microbiol.

[19] Hofmann N, Mwingira F, Shekalaghe S, Robinson LJ, Mueller I, Felger I (2015). Ultra-sensitive detection of *Plasmodium falciparum* by amplification of multi-copy subtelomeric targets. PLoS Med.

[20] Lefterova MI, Budvytiene I, Sandlund J, Färnert A, Banaei N (2015). Simple real-time PCR and amplicon sequencing method for identification of *Plasmodium* species in human whole blood. J Clin Microbiol.

[21] Xu W, Morris U, Aydin-Schmidt B, Msellem MI, Shakely D, Petzold M (2015). SYBR green real-time PCR-RFLP assay targeting the *Plasmodium* cytochrome B gene—a highly sensitive molecular tool for malaria parasite detection and species determination. PLoS ONE.

[22] Koepfli C, Nguitragool W, Hofmann NE, Robinson LJ, Ome-Kaius M, Sattabongkot J (2016). Sensitive and accurate quantification of human malaria parasites using droplet digital PCR (ddPCR). Sci Rep.

[23] Rosanas-Urgell A, Mueller D, Betuela I, Barnadas C, Iga J, Zimmerman PA (2010). Comparison of diagnostic methods for the detection and quantification of the four sympatric *Plasmodium* species in field samples from Papua New Guinea. Malar J.

[24] Lucchi NW, Poorak M, Oberstaller J, DeBarry J, Srinivasamoorthy G, Goldman I (2012). A new single-step PCR assay for the detection of the zoonotic malaria parasite *Plasmodium knowlesi*. PLoS ONE.

[25] Singh B, Kim Sung L, Matusop A, Radhakrishnan A, Shamsul SS, Cox-Singh J (2004). A large focus of naturally acquired *Plasmodium knowlesi* infections in human beings. Lancet.

[26] Haanshuus CG, Mørch K (2020). Detection of remaining *Plasmodium* DNA and gametocytes during follow up after curative malaria treatment among returned travellers in Norway. Malar J.

[27] van Bergen K, Stuitje T, Akkers R, Vermeer E, Castel R, Mank T (2021). Evaluation of a novel real-time PCR assay for the detection, identification and quantification of *Plasmodium* species causing malaria in humans. Malar J.

[28] Beyene MB, Teshome SAY, Terefework Z, Stuitje AR, Abebe T (2022). Assessing the diagnostic performance of a novel RT-PCR fluorescence method for the detection of human *Plasmodium* species. PLoS ONE.

[29] WHO. Malaria Microscopy Quality Assurance Manual, version 2. Geneva, World Health Organization, 2016. https://apps.who.int/iris/handle/10665/204266. Accessed 8 Aug 2022.

[30] Dutch Society for Parasitology. Richtlijn voor de diagnostiek van malaria voor laboratoria in de gezondheidszorg in Nederland. 2009. https://congressus-parasitologie.s3-eu-west-1.amazonaws.com/files/6f2afab3b4174260940b782168777868.pdf. Accessed 12 Aug 2022.

[31] Dutch Society for Parasitology. Voorschriften en gebruik microscoop. Medische Parasitologie, 5th edn. Syntax Media: Utrecht; 2017. p. 289–91.

[32] Padley DJ, Heath AB, Sutherland C, Chiodini PL, Baylis SA (2008). Establishment of the 1st World Health Organization International Standard for *Plasmodium falciparum* DNA for nucleic acid amplification technique (NAT)-based assays. Malar J.

[33] Gimenez AM, Marques RF, Regiart M, Bargieri DY (2021). Diagnostic methods for non-falciparum malaria. Front Cell Infect Microbiol.

[34] Gillet P, Bosselaers K, Cnops L, Bottieau E, Van Esbroeck M, Jacobs J (2009). Evaluation of the SD FK70 malaria Ag *Plasmodium vivax* rapid diagnostic test in a non-endemic setting. Malar J.

[35] Watson OJ, Sumner KM, Janko M, Goel V, Winskill P, Slater HC (2019). False-negative malaria rapid diagnostic test results and their impact on community-based malaria surveys in sub-Saharan Africa. BMJ Glob Health.

[36] Calderaro A, Piccolo G, Montecchini S, Buttrini M, Rossi S, Dell'Anna ML (2018). High prevalence of malaria in a non-endemic setting: comparison of diagnostic tools and patient outcome during a four-year survey (2013–2017). Malar J.

[37] de Gier B, Suryapranata FS, Croughs M, van Genderen PJ, Keuter M, Visser LG (2017). Increase in imported malaria in the Netherlands in asylum seekers and VFR travellers. Malar J.

[38] Berhane A, Anderson K, Mihreteab S, Gresty K, Rogier E, Mohamed S (2018). Major threat to malaria control programs by *Plasmodium falciparum* lacking histidine-rich protein 2, Eritrea. Emerg Infect Dis.

[39] Das D, Price RN, Bethell D, Guerin PJ, Stepniewska K (2013). Early parasitological response following artemisinin-containing regimens: a critical review of the literature. Malar J.

[40] Zwang J, Dorsey G, Mårtensson A, d'Alessandro U, Ndiaye JL, Karema C (2014). *Plasmodium falciparum* clearance in clinical studies of artesunate-amodiaquine and comparator treatments in sub-Saharan Africa, 1999–2009. Malar J.

[41] Dutch knowledge institute of the Federation of Medical Specialists. Richtlijn voor Malaria Diagnostiek Nederland. 2022. https://richtlijnendatabase.nl/richtlijn/malaria_diagnostiek/startpagina_-_malaria_diagnostiek.html. Accessed 9 Dec 2022.

[42] Lalloo DG, Shingadia D, Bell DJ, Beeching NJ, Whitty CJM, Chiodini PL (2016). UK malaria treatment guidelines 2016. J Infect.

[43] Boonstra MB, Koelewijn R, Brienen EAT, Silvis W, Stelma FF, Mank TG (2021). Malaria diagnosis in a malaria non-endemic high-resource country: high variation of diagnostic strategy in clinical laboratories in the Netherlands. Malar J.

[44] Manser M, Olufsen C, Andrews N, Chiodini PL (2013). Estimating the parasitaemia of *Plasmodium falciparum*: experience from a national EQA scheme. Malar J.

[45] Bowers KM, Bell D, Chiodini PL, Barnwell J, Incardona S, Yen S (2009). Inter-rater reliability of malaria parasite counts and comparison of methods. Malar J.

[46] O'Meara WP, McKenzie FE, Magill AJ, Forney JR, Permpanich B, Lucas C (2005). Sources of variability in determining malaria parasite density by microscopy. Am J Trop Med Hyg.

[47] De Koninck AS, Cnops L, Hofmans M, Jacobs J, Van den Bossche D, Philippé J (2017). Diagnostic performance of the loop-mediated isothermal amplification (LAMP) based illumigene® malaria assay in a non-endemic region. Malar J.

[48] Antinori S, Ridolfo AL, Grande R, Galimberti L, Casalini G, Giacomelli A (2021). Loop-mediated isothermal amplification (LAMP) assay for the diagnosis of imported malaria: a narrative review. Infez Med.

[49] WHO. Guidelines for malaria, 3 June 2022. Geneva, World Health Organization, 2022. https://www.who.int/publications/i/item/guidelines-for-malaria. Accesed 15 May 2023.

