# Supplementary material for: Performance of a novel melting curve-based qPCR assay for malaria parasites in routine clinical practice in non-endemic setting
Source: Malar J. 2023 Jun 22;22:191. doi: 10.1186/s12936-023-04617-z (PMC10286418; doi:10.1186/s12936-023-04617-z)
Supplement: Supplementary file 1 — Additional file 1: Fig S1. Melting curves of one P. malariae patient and four P. falciparum (parasitaemia ranging from 0.1-3.1%) patients that were followed after starting anti-malarial treatment. The specific melting curve pattern was used to identify the Plasmodium species. The x-axis shows the temperature (°C). The y-axis shows the negative derivative of fluorescence (RFU) with respect to temperature (T). The Plasmodium species is indicated in the left corner of each figure. The amount of days after the start of anti-malarial treatment is indicated at the right of the melting curves. Red curves correspond to the Texas Red labelled probe, purple curves to the Cy5 labelled probe, and brown curves to the Cy5.5 labelled probe. For the sake of clarity, not all follow-up samples are included in the figures. [file 12936_2023_4617_MOESM1_ESM.docx]

**Performance of a novel melting curve-based qPCR assay for malaria parasites in routine clinical practice in non-endemic setting**

Kim J. M. van Bergen^1*^, Antoine R. Stuitje^2^, Robert C. Akkers^1^, Henricus J. Vermeer^1^, Rob Castel^1^ and Theo G. Mank^3^

^1^Result Laboratorium, Albert Schweitzer Hospital, Albert Schweitzerplaats 25, 3300 AK, Dordrecht, The Netherlands

^2^MRC Holland, Willem Schoutenstraat 1, 1057 DL, Amsterdam, The Netherlands

^3^Regional Laboratory for Medical Microbiology & Public Health, Boerhaavelaan 26, 2035 RC, Haarlem, The Netherlands

*Corresponding author

Email: k.j.m.vanbergen@resultlaboratorium.nl

**Additional file 1: Fig S1**. Melting curves of one *P. malariae* patient and four *P. falciparum* (parasitaemia ranging from 0.1-3.1%) patients that were followed after starting anti-malarial treatment. The specific melting curve pattern was used to identify the *Plasmodium* species. The x-axis shows the temperature (°C). The y-axis shows the negative derivative of fluorescence (RFU) with respect to temperature (T). The *Plasmodium* species is indicated in the left corner of each figure. The amount of days after the start of anti-malarial treatment is indicated at the right of the melting curves. Red curves correspond to the Texas Red labelled probe, purple curves to the Cy5 labelled probe, and brown curves to the Cy5.5 labelled probe. For the sake of clarity, not all follow-up samples are included in the figures**.**

*P. malariae*

Diagnosis

Day 3


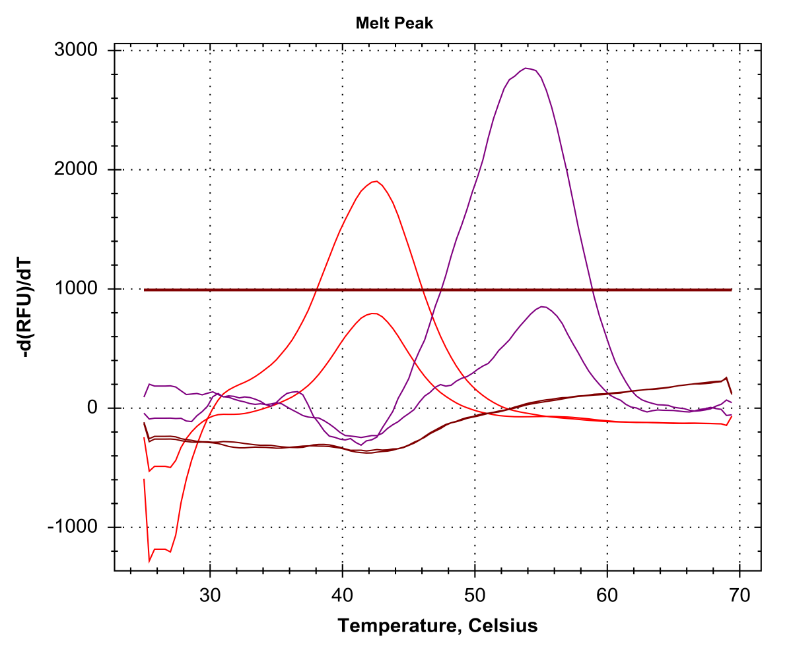


Diagnosis

Day 3

*P. falciparum, 0.1%*


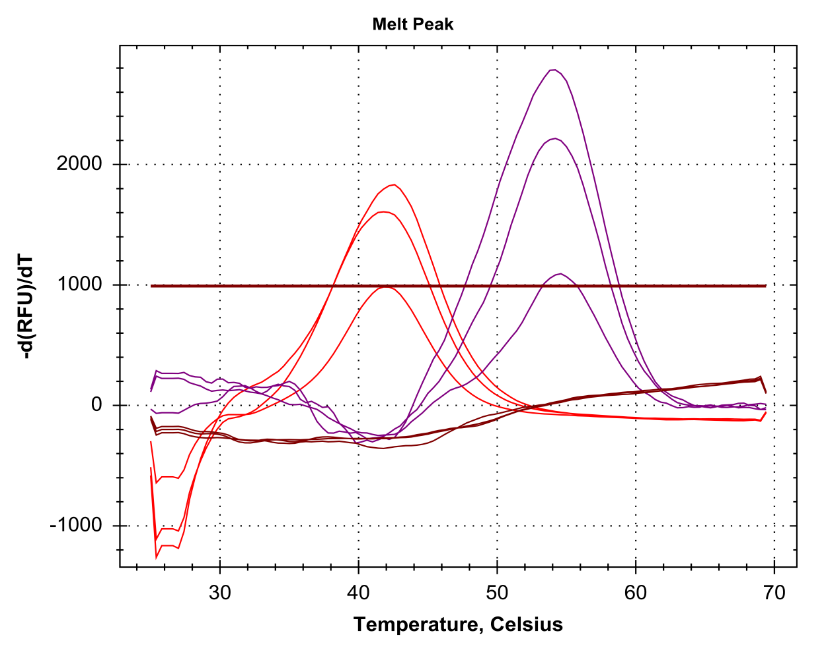


*P. falciparum, 0.1%*

Diagnosis

Day 1

Day 3

*P. falciparum, 0.2%*

Diagnosis

Day 12

Day 1

Diagnosis

Day 8

Day 2

*P. falciparum, 3.1%*
